# Deep learning for DNase I hypersensitive sites identification

**DOI:** 10.1186/s12864-018-5283-8

**Published:** 2018-12-31

**Authors:** Chuqiao Lyu, Lei Wang, Juhua Zhang

**Affiliations:** 1 School of Life Science, Beijing Institute of Technology, South Zhongguancun Street, Beijing, 100081 China; 2 Key Laboratory of Convergence Medical Engineering System and Healthcare Technology the Ministry of Industry and Information Technology, Beijing Institute of Technology, Beijing, China

**Keywords:** DNase I hypersensitive sites, Deep learning, Convolutional neural network

## Abstract

**Background:**

The DNase I hypersensitive sites (DHSs) are associated with the cis-regulatory DNA elements. An efficient method of identifying DHSs can enhance the understanding on the accessibility of chromatin. Despite a multitude of resources available on line including experimental datasets and computational tools, the complex language of DHSs remains incompletely understood.

**Methods:**

Here, we address this challenge using an approach based on a state-of-the-art machine learning method. We present a novel convolutional neural network (CNN) which combined Inception like networks with a gating mechanism for the response of multiple patterns and longterm association in DNA sequences to predict multi-scale DHSs in Arabidopsis, rice and Homo sapiens.

**Results:**

Our method obtains 0.961 area under curve (AUC) on Arabidopsis, 0.969 AUC on rice and 0.918 AUC on Homo sapiens.

**Conclusions:**

Our method provides an efficient and accurate way to identify multi-scale DHSs sequences by deep learning.

## Background

Thirty years ago, it was confirmed that DNA bound by proteins was not degenerated by the DNase I [1]. The early study [2] also showed that there are many highly sensitive nucleotide fragments on the chromosome to DNase I digestion, and they have a high influence on the transcription of the gene. Nucleotide regions that are extremely sensitive to the DNase I are referred to as DNase I hypersensitive sites (DHSs). Some research attempts that DHSs can be precisely coupled with the cis-regulatory elements, including enhancers, promoters, silencers, and locus control regions [3]. Some other research [4, 5] have that many DHSs appear around the highly expressed genes, and few DHSs appear near the low-expressed genes.

Benefit from the improvement of high-throughput sequencing technologies, some new techniques have been applicated to detect DHSs, such as ChIP-seq [6] and DNase-seq [7]. Scientists have detected the DHSs from the human genome and stored them in the public dataset [8]. At the same time, in the field of plant genomes, a large number of DHSs has been detected in plants and be established in a website to visualize these data [9]. There also have a single cell DNase I sequencing (scDNase-seq) [10] method that can identify genome-wide DHSs in a single cell type or less than 1000 cell types. These estimable experimental methods collected many valuable data. It contributes important suggestions for studying the activity of the DNase I, the accessibility of chromatin and gene expression. However, the experimental methods are not only expensive but also takes a lot of time and effort to achieve a complete sequencing, which hinders the progress of subsequent experiments. Covering more and more experimental data, it is still meaningful to design a clever, fast and efficient calculation method to recognize DHSs.

A reasonable dataset of DHSs established and published in 2005, which included 280 DHSs and 737 non-DHSs from erythroid cells [11]. In the next decade, some researchers applied this data to create many useful algorithms for recognize DHSs based on DNA sequences. Support vector machine (SVM) was used to extract dinucleotide features in the sequence [11]. The iDHS-EL [12] use three random forests(RF) to extract different nucleotide sequence features to recognize DHSs. However, due to the imbalance of positive and negative samples, conventional algorithms always get a high false positive rate and not accurate enough for applications. So both gkm-SVM [13] and BIRD [14] use the human genome data to calculate the DNase I hypersensity with regression methods, which have been proved in practice. However, the process of manual feature extraction and design is relatively complex, which requires a lot of patience, and is not conducive to the generalization of the model. As everyone knows, DHSs are both tissue-specific and cell-specific. It was reported that 34% of human DHSs were specifically appear in one cell line, 66% were appear in both cell lines, and only 0.09*%* can be detected in all cell lines, analyzing high throughput sequencing results of 125 human cell lines [8]. The proportion of DHSs in human exons is only 1/2 of rice [15]. However, both of their proportion of DHSs in the intergenic region is coincident [16]. In other words, the activity of DHSs is closely related to the epigenetic factors. In Arabidopsis thaliana, once a hypermethylated DNA fragment loses its methylation, the sensitivity of DNase I will be greatly increased [17]. Histone modifications also affect chromatin sensitivity of DNase I in varying degrees [18–21]. The previous calculation methods have obtained less than ideal results, due to these fundamental reasons, and it could be almost impossible that the accuracy rate of recognition of DHSs were further improved with previous algorithms simply based on DNA sequences in a single cell type.

In order to avoid the limitations of the artificial feature, we try to use the deep learning algorithm [22] to actualize the classification of DHSs and turn in the direction of DHSs combined in a large number of cell types. The deep learning algorithm has unique advantages in feature extraction, even if it can explore some features that cannot be visualized by the original data. For example, in the field of natural language processing (NLP), recurrent neural network (RNN) [23] can mine contextual information [24] from a text, understand the emotions it expresses, and even answer the questions [25]. In the field of image recognition, convolutional neural network (CNN) [26] can understand the pixel value from the local to the whole image and accomplish detection and segmentation [27] of the target. It is very different from other statistical analysis methods. In deep learning models, the network structure is established to complete the understanding of the original data layer by layer, and both of feature extraction and classification in models are completed automatically.

But deep learning also has its weaknesses. Firstly, the structure of the model is a black box which cannot be described. Secondly, a large number of labeled data supplies are required during the training process of a supervised model. But considering the unique expressiveness of deep learning algorithms, they are still excellent choices in all existing calculation methods. In recent years, the deep methods has granted the computational power to resolve genomics research questions. Some researchers [28] have proved the validity of CNN, RNN and their mixture models in gene sequence classification. DeepBind [29], DeepSEA [30] and Basset [31] used CNN to predict protein binding sites, non-coding regions and the functional activity of DNA sequences, respectively. ProLanGO [32], DeepNano [33] and DanQ [34] used RNN to predict protein expression, base recognition and non coding DNA, respectively. Deep GDashboard [28] and BiRen [35] used the CNN-RNN hybrid framework to predict the locations and enhancers of transcription factor binding, respectively. All of these methods have achieved good results. RNN can recognize different length sequences according to its loop structure and understand the characteristics of long-term association. However, it can not carry out parallel computing, which needs a lot of time for training. CNN can only handle the sequence of fixed length and broken segments, but it runs fast. Most of the hybrid architectures only stacks the CNN and the RNN, without considering combining the advantages of them. So here we make a new model that combines the speed advantages of CNN and effectively understands the long-range association of sequences, to support training of indefinite long sequence, and we established Arabidopsis, rice and Homo sapiens datasets to verify our model. Finally our model achieved state-of-the-art results on the datasets of Arabidopsis and rice, also achieved ambitious results on Homo sapiens.

## Methods

### Model establishing

Because of the novel gate layer of LeNup [36], it is straightforward to learn the association of the long ranges of nucleotide fragments. We contemplated that all the active DNA fragments have three-dimensional structures. With the predominant feature extraction capability of CNN network, the entry gate control can make the organization of DNA in the three-dimensional structure to a feature. So LeNup has a good performance in nucleosome positioning. We also made experiments shown in Table 1, that the gate layer structure is still valid in DHSs. It indicates that the design of DNA recognition by gated layers is effective. So, on the basis of the DHSs classification model, we fine-tuning the first five convolution layers of LeNup and then changing the last pooling layer to special pyramid pooling (SPP) layer. Finally, we uses the LeakyReLU function to activate the entire network. The most important of these adjustments is the SPP layer, which enables the model to support the variable-length nucleotide segments as input in a reasonable range, while the other adjustments are designed to prevent the gradient disappearance and improve the speed of training.

**Table 1:** Comparison of DHSs predictions in different species

Species	Interval(bp)	*S*_*n*_(*%*)	*S*_*p*_(*%*)	*ACC*(*%*)	*MCC*	*AUC*
Arabidopsis	50	90.64	90.78	90.70	0.813	0.961
	100	88.27	92.94	90.46	0.810	0.956
	200	86.23	93.54	89.66	0.796	0.953
Rice	50	89.30	94.44	91.87	0.838	0.969
	100	89.88	93.68	91.78	0.836	0.962
	200	82.73	95.66	89.19	0.790	0.959
Human	50	86.99	85.31	86.15	0.723	0.918
	100	82.26	88.73	85.51	0.711	0.911
	200	77.31	89.96	83.65	0.678	0.849

**Fig. 1:**
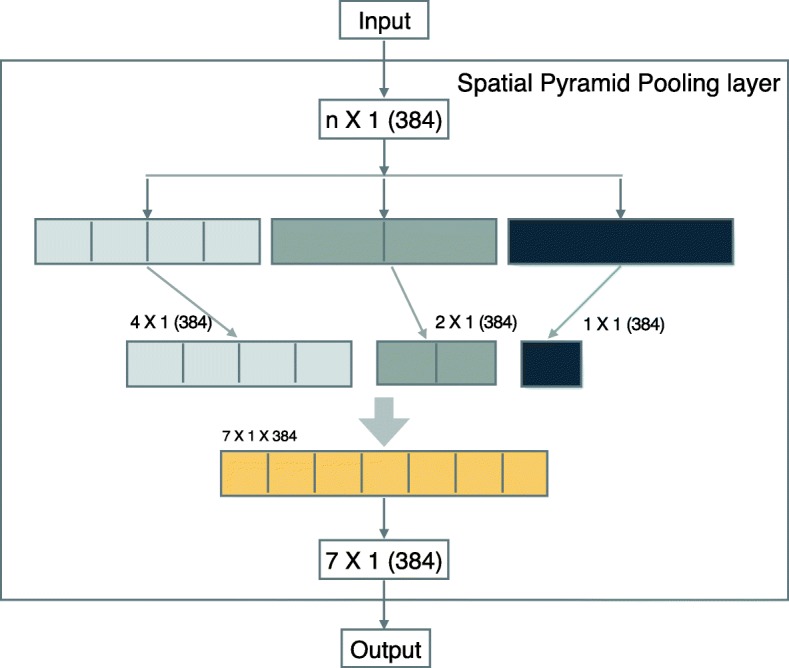
The internal structure of the SPP layer: Number of 384 describes the number of the fifth convolution layer. The fifth layer convolution is the last layer. The output obtained from the fifth layer convolution layer are pooled through the pooling layer of *n*/4, *n*/2, and *n*/1 dimension respectively, then the 4, 2, and 1 features are obtained. Finally, the 4+2+1=7 features are gathered into the full connection layer

**The special pyramid pooling layer:** The DHSs are variable-large-length nucleotide segments (from tens bp to thousands of bp). Deep learning models normally support a fixed-length input (LeNup network only supports a 147bp length of nucleotide segments). Because, first of all, the convolution layer is insensitive to the scale of input as long as the scale does not exceed the computation range. But the output of convolution layer needs to pass through the full connection layer, where the connection parameters are fixed. In the field of image recognition, the usual way to solve this problem of multi-scale input is to normalize the pictures to the combined dimension by scaling and clipping. However, the nucleotide sequences are different with the images. Because the length of DHSs is longer than the wide of an image, and the initial information will miss if we cut the nucleotide sequence. Therefore, we added the SPP layer between the convolution layer and the full connection layer of LeNup in order to allow the models to operate the DHSs sequence information. This method (first proposed in 2015 [37]) was used to solve the problem of multi-size of images in CNN. SPP layer applies several multi-size pooling layers to replace one pooling layer between the last convolution layer and the first full connection layer. Firstly, in this paper, we encoded the DNA sequences to the one-hot numbers, whose fragment was converted into a two-dimensional matrix of *n*×4. The DNA sequence is similar to the multi-scale image, but the DNA encoding only changes one dimension (length), while the image is changed in two dimensions (wide and height). Secondly, we used the SPP layer separates the output which from the last convolution layer into 1, 2, and 4 parts, and recorded the average value of every part. Finally, we stacked all values as the output of the SPP layer. Through the SPP layer, the dimension of uncertain *n*×4 (*n*>4, in this article) of output can be modified into 7×4 (1+2+4=7), and then the fixed size output can enter the full connection layer to do classification prediction. The structure of the SPP layer is shown as illustrated (Fig. 1).

**The leaky rectified linear unit**[38]: It shows that the number of convolution layers of CNN model is positively related to the performance. If the model is deeper, the problem of gradient disappearance is more serious. Considering the nucleotides have less information than words or pixels (only four nucleotides of A, C, G, and T will be used), the problem of gradient disappearance is particularly visible when the DHSs classification model is trained. Here we desire that the gradient of the model does not disappear when the model with five convolution layers is being trained. So we used the leaky rectified linear unit (LeakyReLU) [39] to fix this problem. The LeakyReLU function is mathematically given by:

1 $$ y_{i} = \left\{ \begin{array}{ccc} x_{i} & if & x_{i} \ge 0,\\ \frac{x_{i}}{a_{i}} & if & x_{i} < 0. \end{array} \right.  $$

*a*_*i*_ indicates the range correction parameter (this model takes 100.0). *x*_*i*_ indicates the input of LeakyReLU layer, and *y*_*i*_ indicates the output after activation. To a certain extent the LeakyReLU layer can effectively prevent the gradient from vanishing. Although the LeakyReLU layer cannot provid a striking increase in accuracy of prediction, but the problem of gradient vanishing was effectively prevented during the training process, and the robustness of the model was increased.

**Fig. 2:**
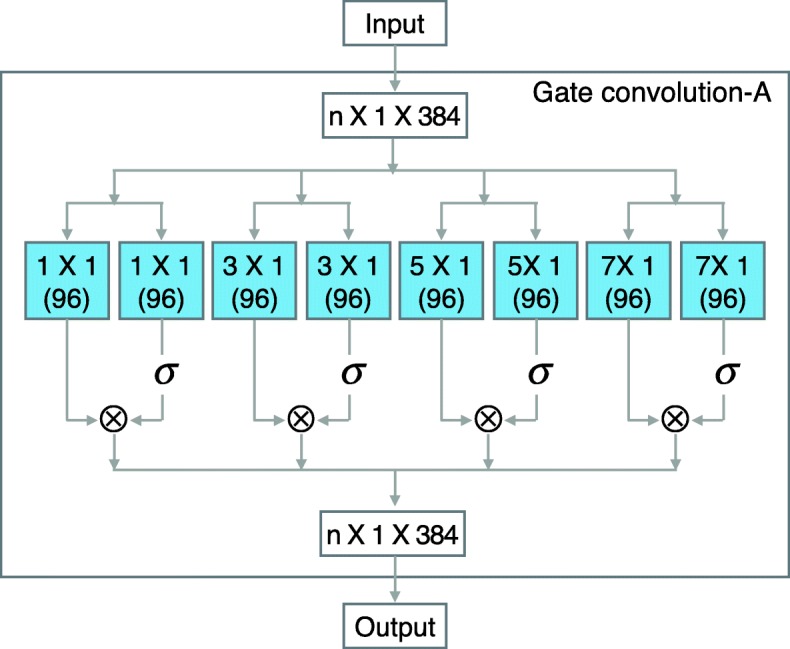
Gated Inception-A block

**Fig. 3:**
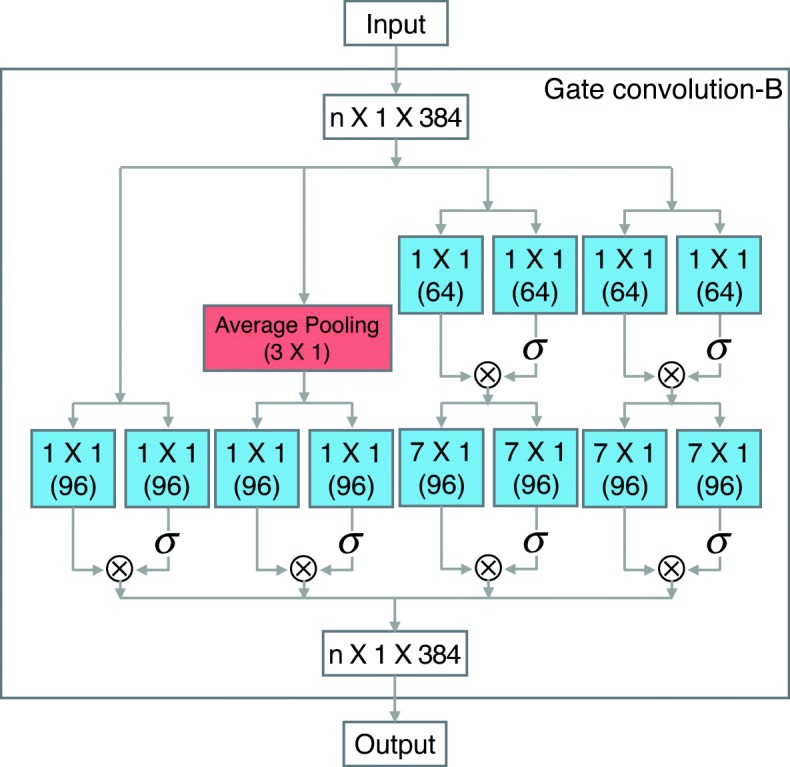
Gated Inception-B block

**Fig. 4:**
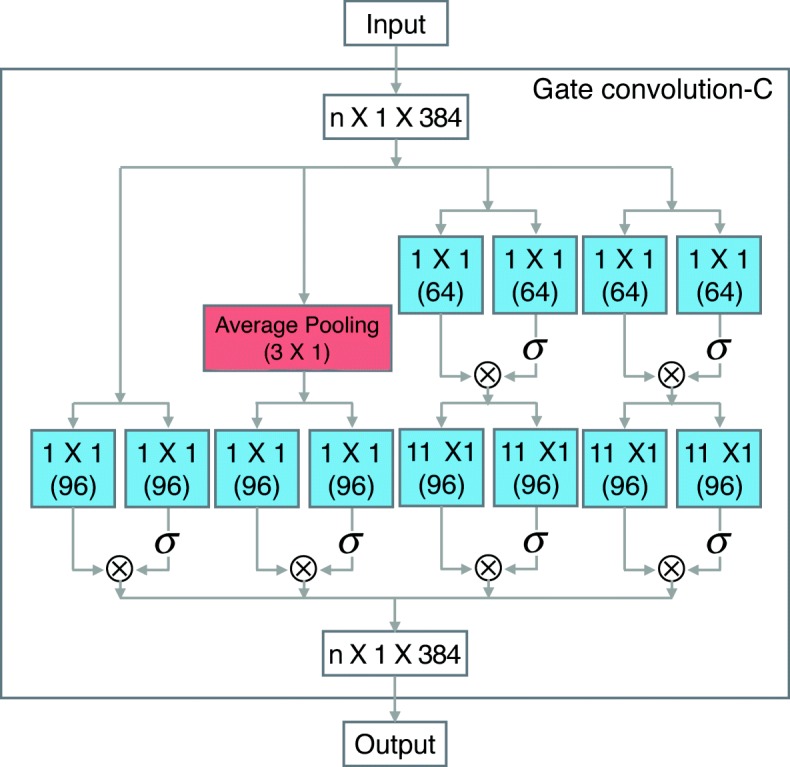
Gated Inception-C block

**Fig. 5:**
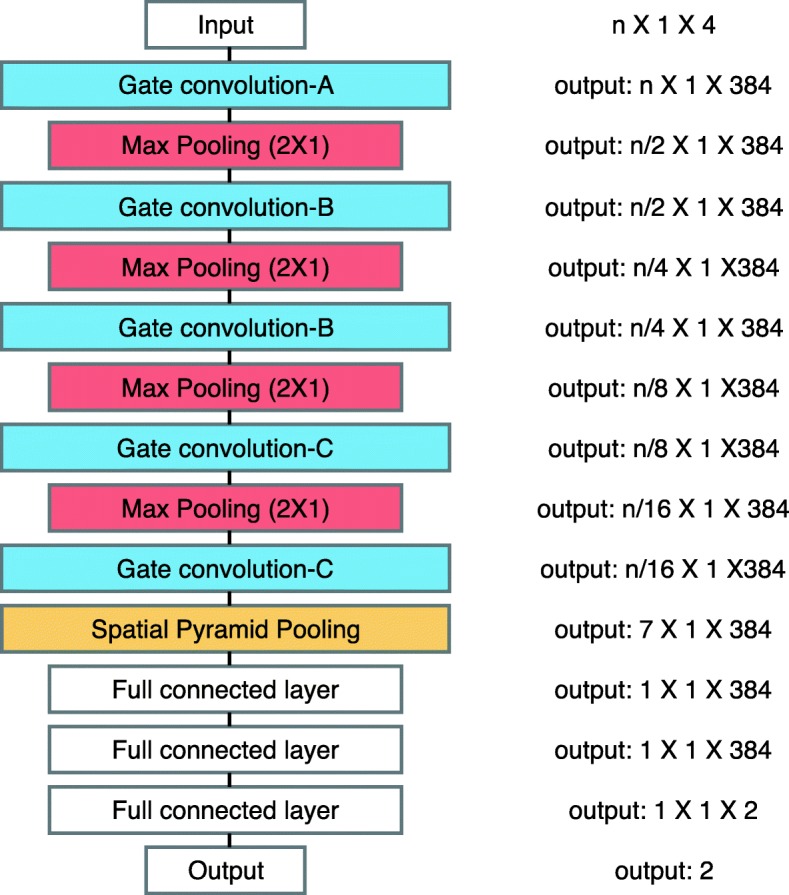
The figure shows that the overall construction of the model, which including 5 convolution layers, 4 maximum pooling layers, 1 SPP layer, and 3 fully connected layers. In addition to these visible structures, every layer is activated by LeakyReLU and followed by the dropout layer (the parameter is 0.3), and each fully connected layer is normalized by the batch normalization (BN, whose parameter is 0.5), which can speed up the convergence of the network. Dropout layers and BN layers are not depicted in the diagram

**Other layers:** In addition to modifying the pooling layer and the activation function, we also fine-tuning the convolution layers of LeNup. The structural parameters of each layer are depicted in Figs. 2, 3, 4, we add 1, 3, 5, and 7 filters in the Gate-inception-A. The Gate-inception-B and the Gate-inception-C module are consistent with LeNup. Every convolution kernel of the model gets a gated convolution operation. The model is finally depicted in Fig. 5.

**Fig. 6:**
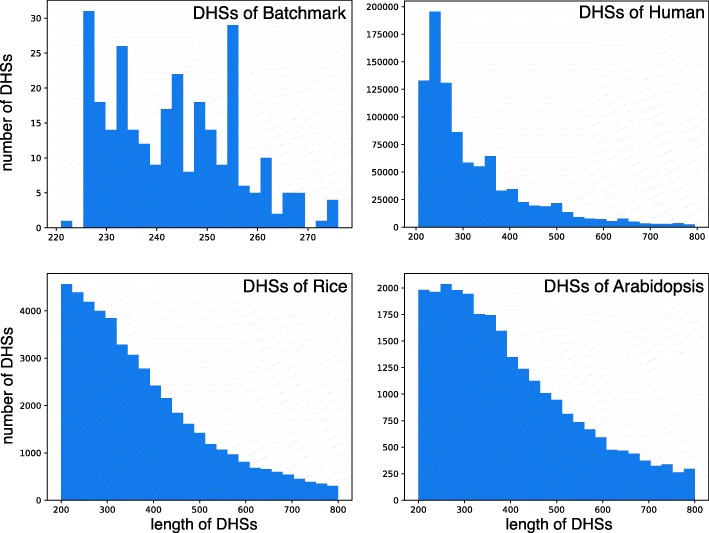
Analysis of Arabidopsis, rice, and human data shows that the number of DHSs decreases with increasing length. However, in the benchmark dataset, because of the tiny size, it cannot display the trend. It is embarrassing to enhance the prediction capacity of the model, so it is easy to fall into overfitting

**Fig. 7:**
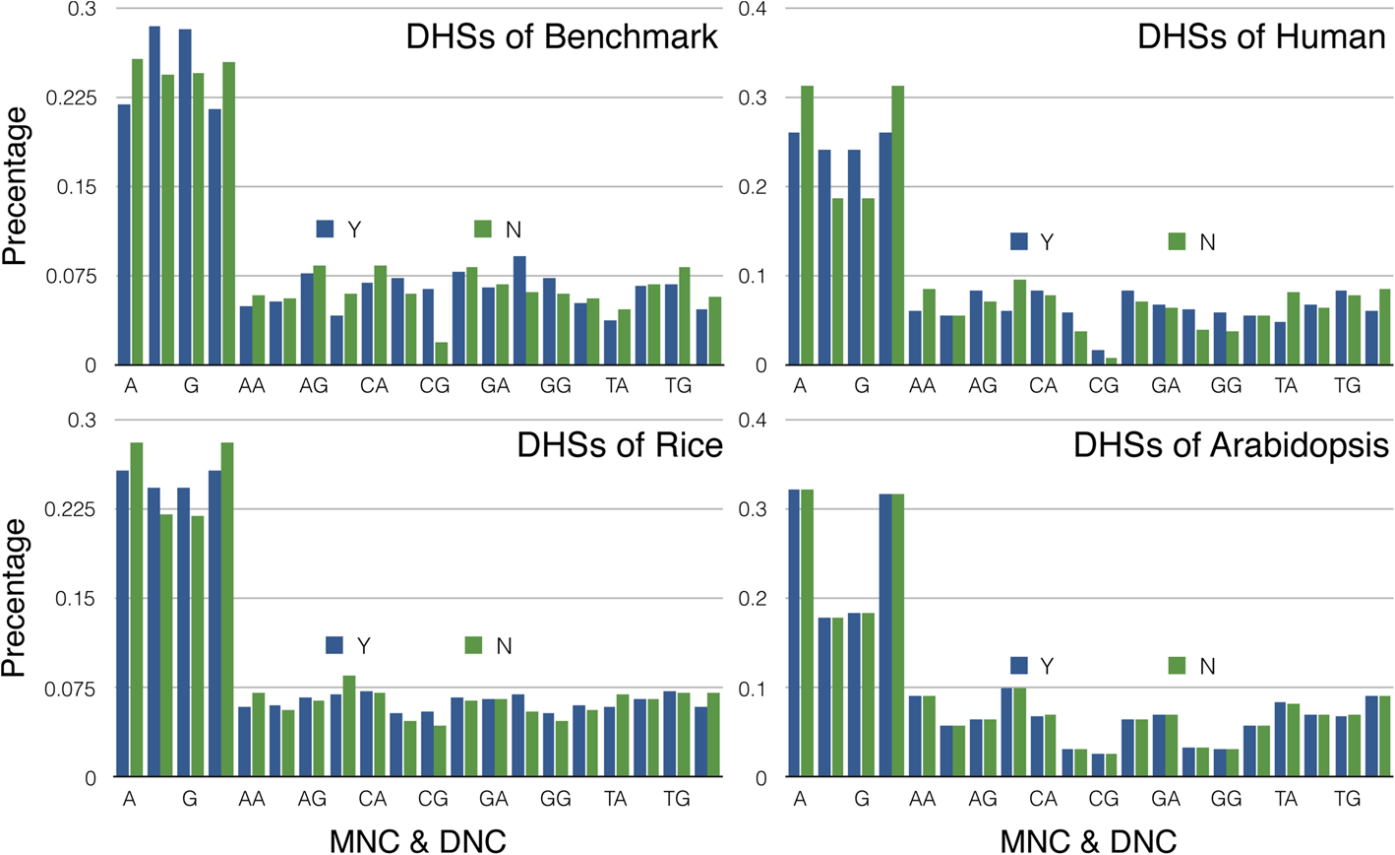
It is obvious from the graph that the difference of nucleotide ratios between DHSs and non-DHSs will decreases with reducing of the complexity of species. Benchmark dataset quiet has a weak coverage of sample space, because of the small amount of data. At the same time, a larger number of non-DHSs (benchmark dataset has 280 DHSs and 737 non-DHSs) were more likely to lead to overfitting of the model on the non-DHSs. It can also be seen that there is little distinction the proportion of MNC and DNC in arabidopsis’s DHSs and non-DHSs, which indicates that it is challenging to use the feature of nucleotide site training model on the benchmark dataset

**The establishment of data sets:** After setting up the training model, we need the appropriate datasets to test its performance. Firstly, we downloaded the DHSs data of aiabidopsis and rice from the website (http://www.plantdhs.org/Download). The DHSs data of human was also obtained from ENCODE. The different species have different length distribution of DNA fragments, and the expression level of the DHSs in the different cell lines of one specie is different. So we used the range of DHSs derived from all the DHSs known in the whole genome of the species. Secondly, in order to ensure the stability of the model, we used the length from 200 to 800bp for each DNA fragments. At the same time, we selected an equal length DNA fragment for each DHSs in the non-DHSs region of the same chromosome as a negative sample. The length distribution of the non-DHSs in the obtained data is exactly same as that of the DHSs. Finally, we used cd-hit [40] software to remove the higher identity sequence in both positive and negative samples. Through these methods, we setted up three datasets of Arabidopsis, rice and Homo sapiens respectively (Table 2). All of the data are only selected from euchromosomes. At the same time, in order to test the reliability of datasets, we accompanied these three datasets with benchmark datasets [11]. The results are shown in Figs. 6 and 7.

**Table 2:** The statistical results of three datasets

Species	Positive(P)	Negative(N)	Avg(bp)	Ratio(P:N)
Arabidopsis	26399	23112	403	1.14
Rice	56033	46201	376	1.21
Human	943681	742476	326	1.27

### Multi-scale training strategy

**Fig. 8:**
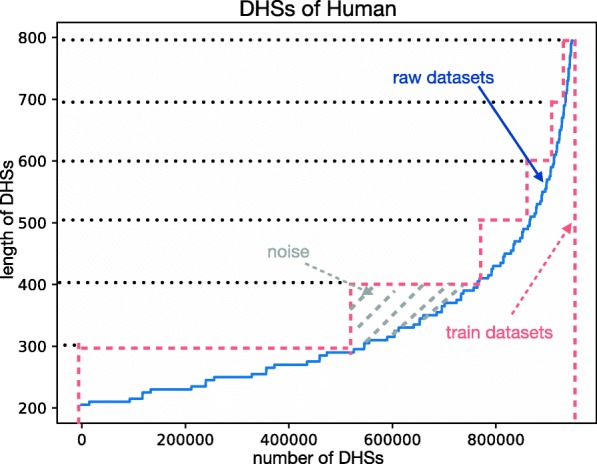
The figure above shows the ratio of the training data to the non-extended data. Firstly, the DHSs is sorted according to the length of the fragments. The vertical axis indicates the length of each fragment. The horizontal axis indicates the number of the fragment in the entire dataset. The blue line indicates the non-extended data, and the area underneath it indicates the number of bases in the whole non-extended data. The red dotted line indicates the training data, whose bottom area indicates the base number of the whole training data. The gray area indicates the extra fragments, which is also used to speed up training and extended by us

**The distribution of datasets:** We used random extraction and cross validation method to partition training datasets and testing datasets. 5-fold cross validation was used in both Arabidopsis and rice, and 10-fold in Homo sapiens. Because the number of Homo sapiens DHSs was too large, so the ratio of training data and testing data was bound to 1:9. Our original purpose was that the model can receive multi-scale input during the training process. Theoretically, the above model can accept input from arbitrary dimensions. However, the graphic processing unit (GPU) in computers can only receive fixed-length inputs when parallel computing. Here we hope to give full play to the advantages of GPU. So during the training process, we divided the training data into multiple parts of the training data in accordance the range of their length (for example, the length of datasets will be divided into 200bp-400bp, 400bp-600bp, and 600bp-800bp of the length according to the interval of 200bp). Since each data was selected from the true existing chromosomes (whether it is a positive or negative sample), we extended it into the longest BP from both ends (for example, we extend all the length of the DHSs in the 200bp-400bp part to 400bp length). From this way, we got many new DHSs datasets, but the length of nucleotide sequences in each dataset was same, and the ratio of positive and negative samples was approximately 1:1. It was also possible to ensure that there was a complete DHS in every positive sample after extension. The advantage of this method is that the length of the sequence extended to two ends can be controlled by ourself. And the training datasets will be amplified if we change the length of the left or right extensed fragments. (It depending on the number of samples, the number of Arabidopsis training data was amplified by 3 times, and the number of rice and the number of Homo sapiens are sufficient, so they didn’t be expanded). Training with the amplified data can increase the accuracy on the testing data (give an average increase of about 1%). But it also introduced some noise in the positive sample. Therefore, in order to get faster convergence speed, it is unavoidable to sacrifice the accuracy of some models. However, it should be noted that the fragment length of the testing data was still arbitrary, and the quality of the model was only determined by the performance on the testing. The training datasets selection method and the generated noise are shown in Fig. 8 (we only select the human DHSs at intervals of 100bp as example).

After filtering, We got several datasets of various scales. In training phase, these training sets share the same parameters of one model. Because of the presence of SPP layer, the dimensions of parameters in the model will not change. Here referring to the multi-scale training method [37]. For example we took the interval as 200bp to split the training datasets. So we obtained three training datasets which had the lenght of 400bp, 600bp and 800bp respectively. Firstly, we initialized the network parameters, and the 400bp’s set was used to train a complete epoch. Secondly, we retained the parameters of the model, and trained a complete epoch with 600bp’s set. and then 800bp’s. When all data was trained once, it would be recorded as a complete iteration. This training method allows the model to learn input information from different dimensions, and retain the advantages of GPU. During the training process, we found the convergence rate of the segmented training loss was similar from the single length training, and only cost a slight time in the process of converting input length.

### Training parameters

We have trained our models running on a single NVidia Quadro P6000 with stochastic gradient descent with momentum in pytorch. PyTorch is a handy deep learning library that extends Python. The training step used momentum with a decay of 0.98, a learning rate of 0.002, and decayed every epoch using an exponential rate of 0.97. We also used a mini-batch size of 128 samples and trained the model for 100 iterations. Each iteration taked about one minute. The well trained model size was about 12.5 megabyte, and the number of parameters was 3,077,382.

## Results

We selected the sensitivity (Sn), the specificity (Sp), the accuracy (ACC) and the Matthew’s correlation coefficient (MCC) to evaluate the analogous method. These were generally used in identifying the consequences of models. They are defined as follows:

2 $$ \begin{aligned} S_{n}&=\frac{T_{p}}{T_{p}+F_{n}} \\ S_{p}&=\frac{T_{n}}{T_{n}+F_{p}} \\ ACC&=\frac{T_{p}+T_{n}}{T_{p}+F_{n}+T_{n}+F_{p}} \\ MCC&=\frac{T_{p}\times T_{n}-F_{p}\times F_{n}}{\sqrt{(T_{n}\,+\,F_{n})\times\!(T_{n}+F_{p})\times\!(T_{p}+F_{n})\times\!(T_{p}+F_{p})}} \end{aligned}  $$

### Multi-scale training in different species

**Fig. 9:**
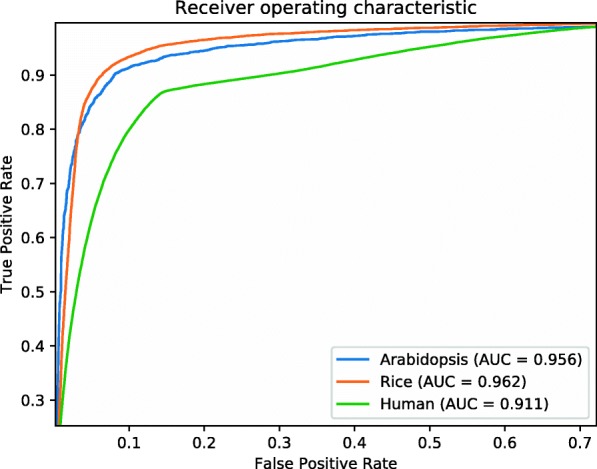
Two ROC curves obtained from 5-fold cross-validation tests using the genome dataset of Arabidopsis, Rice; One ROC curve obtained from 10-fold cross-validation tests using the genome dataset of Human

In order to verify the best segmental training effect, we tested the interval of 50bp, 100bp, and 200bp to divide the training data, and evaluated the accuracy on the testing data of Arabidopsis, rice and human respectively. The ROC curves are shown in Fig. 9. After testing, it is easy to find that reducing the interval in three species sets can improve the accuracy of the model. With the increase of intervals, the proportion of noise also increase, which lead to the bias of the model lean to negative samples. It is shown in the Arabidopsis dataset that *S*_*n*_ decreases and *S*_*p*_ increases with increase of interval. This indicates that the noise can lead to over fitting of models on negative samples. But the lower interval can not get better results on all evaluation indicators. There is not a large difference between the 50bp interval and the 100bp interval training method on rice. For Homo sapiens, the 50bp interval brought a very low AUC. This indicates that the model may incline to positive samples. Therefore, the synthesis of the three datasets proves that there is a reasonable and no lose of model capability to divide datasets with 100bp interval. At the same time, in the process of cross validation, we got a very stable rate of accuracy on each testing data. Arabidopsis got 90±2*%*, rice got 91±2*%*, and Homo sapiens got 86±2*%*. The compared results are shown in Table 1.

### Performance comparison with single-scale training

In order to compare with other methods, we referred to other methods [41]. The author had done a lot of work on the choice of non-DHSs, and they also had established a DHSs classification model based on their database called pDHS-ELM. We used their datasets which can be downloaded from the website (https://github.com/wesd778/dhsNet/tree/master/raw) to train our model. However, it is important to note that the author chooses the non-DHS locus randomly in 100bp-600bp, which makes the length distribution of negative samples inconsistent with positive samples. If we use the multi-scale training method, the proportion of negative samples will be very high (For example, in 500bp-600bp, the number of non-DHSs will ten times with DHSs). Therefore, in order to compared the reasonable experimental results, we gave up the multi-scale inputs and extended all the DNA sequences to 600bp. The training method also uses 5-fold cross validation, and without completely changing the structure and training parameters of the model. The final results are listed in Table 3. We also downloaded the single-scale (600pb) human dataset in the published research using Basset [31]. Basset had three convolution layers and two fully connected layers, which was powerful in DHSs identification. We got the mean-auc value of 0.890 (0.780 for gkm-svm, 0.895 for basset) without completely changing the structure and training parameters of the model, which was slightly worse than that of multi-scale training (0.918, Table 1). It also proves that the method of multi-scale training of DHSs is effective.

**Table 3:** Our mothod performance measured by 5-fold cross validation

Methods	*S*_*n*_(*%*)	*S*_*p*_(*%*)	*ACC*(*%*)	*MCC*
SVM-Revchmer [42]	82.54	79.78	81.66	0.634
PseDNC-SVM [43]	81.30	78.91	80.11	0.602
iDHS-EL [44]	81.24	76.11	78.61	0.572
Unb-PseTNC [45]	86.48	83.74	85.11	0.702
pDHS-ELM [41]	89.17	87.78	88.48	0.717
ours	88.25	96.49	92.88	0.856

Note: The datasets were downloaded from [41]

## Discussion

After comparison, we found that the new network structure shows a surprising result on a single-scale dataset. It also proved that given too much emphasis on the proportion of single nucleotides or polynucleotides in DNA fragments would make a large limitation on the results of model. By combining the gate layers and the inception layers in deep learning model, the features of the DHSs could be more accurately captured. In a sense, it was very similar to sentiment analysis in natural language processing (NLP).

## Conclusions

The experimental conclusions illustrate that CNN network can effectively extract features from nucleotide sequences and be used for genome-wide DHSs classification. We can not prove that the DHSs are completely related with DNA sequence, because they have specific expression in different cell lines. However, as a result, the new model can be used as a tool for detecting DHSs, only to give the sequencing data of the corresponding cell lines and the DHSs from it for training. After the model converges, the nucleotide fragments in the same cell line can be assessed in a very powerful accuracy rate. Moreover, based on this model, it produces a good solution for the problem of DNA segment classification with uncertain length. If there are provide adequate datasets, such as regulatory units, cancer genes, and so on, we believed that the sequence-based flexible classification model will be more widely used.

## Abbreviations

ACC: Accuracy
AUC: Area under curve
CNN: Convolutional neural network
DHSs: DNase I hypersensitive sites
GPU: Graphic processing unit
LeakyReLU: Leaky rectified linear unit
MCC: Matthew’s correlation coefficient
NLP: Natural language processing
RNN: Recurrent neural network
Sn: Sensitivity
SPP: Special pyramid pooling
Sp: Specificity
